# Magnitude of impaired fasting glucose and undiagnosed diabetic mellitus and associated risk factors among adults living in Woreta town, northwest Ethiopia: a community-based cross-sectional study, 2021

**DOI:** 10.1186/s12902-022-01156-7

**Published:** 2022-10-05

**Authors:** Assefa Agegnehu Teshome, Shegaw Zeleke Baih, Amare Kassaw Wolie, Misganaw Asmamaw Mengstie, Zelalem Tilahun Muche, Shambel Nigussie Amare, Mohammed Abdu seid, Getachew Yideg Yitbark, Yalew Melkamu Molla, Nega Dagnaw Baye, Gashaw walle Ayehu

**Affiliations:** 1 Department of biomedical science, college of health science, Debre Tabor University, Debre Tabor, P.O. Box 272, Ethiopia; 2 Department of adult health nursing, college of health science, Debre Tabor University, Debre Tabor, Ethiopia; 3 Department of pediatrics and child health nursing, college of health science, Debre Tabor University, Debre Tabor, Ethiopia; 4 Department of clinical pharmacy, school of pharmacy, college of health and medical science, Haramaya University, Harar, Ethiopia; 5 Department of pediatrics and child health, college of medicine and health science, university of Gondar, Gondar, Ethiopia

**Keywords:** Impaired fasting glucose, Undiagnosed diabetic mellitus, Risk factors, Ethiopia

## Abstract

**Background:**

Impaired fasting glucose (IFG) is an early warning system that provides prior information to prevent the future development of DM and diabetes-related problems, but early detection of DM is not practically applicable in Ethiopia. This study was aimed to assess the magnitude of impaired fasting glucose and undiagnosed diabetes mellitus (DM) and associated factors.

**Methods:**

A community-based, cross-sectional study was conducted from May to June 30, 2021. A structured interviewer-administered questionnaire was used to collect data. Anthropometric measurements were also recorded. A fasting blood sugar (FBS) test was assessed by samples taken early in the morning. Epi-Info 7.2.5.0 was used to enter data, which was then exported to SPSS 25 for analysis. To identify factors associated with IFG, logistics regression was used. The level of statistical significance was declared at p 0.05.

**Result:**

Three hundred and twenty-four (324) participants with a mean age of 43.76 ± 17.29 years were enrolled. The overall magnitude of impaired fasting glucose (IFG) and undiagnosed diabetes mellitus (DM) were 43.2% and 10.0%, respectively. Waist circumference (AOR: 1.72, 95% CI 1.23–3.14), hypertension (AOR: 3.48, 95% CI 1.35–8.89), family history of Diabetic mellitus (AOR: 2.34, 95% CI 1.37–5.79) and hypertriglyceridemia (AOR: 2.35, 95% CI 1.41–5.43) were found to be independently associated with impaired fasting glucose.

**Conclusion:**

Individuals who are overweight, hypertriglyceridemia, and are hypertensive should have regular checkups and community-based screening.

## Background

Diabetes mellitus (DM) is a public health problem characterized by a high blood sugar level in the body. It can be caused by a lack of either insulin secretion or insulin resistance. Its magnitude has been increasing in recent decades. The magnitude in people above the age of 18 increased from 4.7% to 1980 to 8.8% in 2017 [1–3]. It affects more than 425million people worldwide, with the number expected to rise to 529million by 2030 [1].

Diabetes mellitus is a big public health problem with serious consequences. Undiagnosed diabetes mellitus (UDM) affects 50% of people aged 20 to 79 years in the world, 69% in Africa, which is nearly double that of high-income nations (37%). This provides new information on Africa’s high rate of sickness and mortality, which could occur at a younger age [2, 3].

Impaired fasting glucose (IFG) is characterized by glycemic levels that are greater than normal but below diabetes thresholds (fasting glucose > 60.0 mmol/L and > 70.0 mmol/L). It is a major risk factor for diabetes and its consequences, including nephropathy, diabetic retinopathy, and an elevated risk of macrovascular disease [4–6]. As a result, understanding IFG is critical for avoiding diabetes forecasts in the future.

According to studies in Behar Dar city and rural areas of Dire Dawa, UDM was 10.2% and 6.2%, respectively [7, 8]. A worldwide study by 2030 reported that 470million people will be affected by IFG [9, 10]. In addition, studies have shown that people diagnosed with IFG are more likely to develop diabetes. Diabetes is a 5–10% annual risk for patients with IFG, according to Tabak et al. [11]. Larson et al. found that 25% of postmenopausal women with impaired fasting glucose (IFG) or impaired glucose tolerance (IGT) tests progressed to type 2 diabetes (T2DM) in 5 years [12]. However, there is evidence that controlling IFG with lifestyle changes, including physical activity and healthy diets, [13–15], can prevent or postpone the advancement of DM, and another study found that behavioral changes alone lowered the risk of DM by 40–70%[11]. In general, it is critical to identify people who have an IFG condition so that early preventive actions can be implemented.

In 2015, the International Diabetes Federation (IDF) reported that the magnitude of DM in Ethiopia was 2.2%[16]. Despite the fact that IFG is an early warning system that provides a warning to prevent the future development of DM and diabetes-related problems, data on the prevalence of prediabetes (IFG), undiagnosed DM, and associated risk factors in Ethiopia is inadequate. As a result, this research will give baseline data on the magnitude of IFG, UDM, and associated factors in Ethiopia.

## Methods

### Study area

The research was carried out in Wereta, Fogera district. Woreta town is located in the Amhara National Regional State, 561km from Addis Ababa, the capital city of Ethiopia. The town has 18,400 inhabitants, living in five kebeles (the smallest administrative units). There is one government health center and one private hospital in town.

### Study design and period

A community-based cross-sectional study design was conducted from June to July 2021.

### Source population

All adults (age over 18 years old) who live in Woreta town.

### Study population

Adults who live in Woreta town (selected kebeles) and fulfill the inclusion criteria.

### Sample size

The sample size was generated using the single population proportion approach, which factored in a 12% prevalence of IFG discovered in a prior study in Koladiba town, northwest Ethiopia [17], a 5% level of significance, and a 5% margin of error. The minimum sample size was 324 as a result of multiplying the estimated result (162) by the design effect of 2.

### Sampling procedure

A multistage sampling procedure was used to choose the participants, and three kebeles (the smallest administrative units with similar demographics) were picked at random from five kebeles. Participants were proportionally assigned to each of the selected kebeles based on the number of households. Thus, 120, 98, and 106 participants were selected from Kebele one, three, and five, respectively, using the systematic random sampling technique. When there were multiple eligible subjects in a family, the lottery method was employed to choose one at random.

## Data collection procedures

Before the actual data collection, the data collectors received a 3-day training on interviewing techniques, questionnaire administration, and physical measuring procedures. Data was collected by two health officers and two laboratory technicians.

The timing of a participant’s last meal was checked to ensure that they had fasted for at least eight hours the night before. A pre-test was conducted from non-study areas to confirm that the data collectors and responders understood the questions. As a result of the pre-test comments, necessary improvements were made prior to actual data collection.

The WHO NCD STEPS tool, which consists of three steps for measuring the risk of NCD risk variables, was used in this study [18]. First, the study population’s core and expanded socio-demographic and behavioral characteristics are gathered. The second step involves taking core and expanded physical measurements, while the third step involves biochemical analysis [18]. The data collection application was originally written in English and then translated into Amharic, the commonly spoken language in the study area, by language experts. To guarantee uniformity, a back translation into English was undertaken prior to data collection.

### Variable measurements

The International Diabetes Association’s (IDA) definition was used to define DM: “fasting blood glucose (FBG) ≥126mg/dL. Previously undiagnosed DM was defined as participants who had not had their blood sugar tested before and were not taking DM medications during the survey and had an FBG ≥126mg/dL“[16].

Anthropometric measures were taken using standardized methodologies and calibrated equipment. Each study subject was weighed to the nearest 0.1kg. Height and weight were measured to calculate their BMI (kg/m2). Height was measured using a portable stadiometer. “Underweight was defined as < 18.5 kg/m^2^, normal was defined as 18.5–24.9 kg/m^2^, overweight was defined as 25–29.9 kg/m^2^, and obesity was defined as ≥ 30 kg/m^2^”. The midpoint between the lower margin of the least palpable rib and the top of the iliac crest was marked to measure the waist circumference (WC). According to the World Health Organization, WC values of more than 102cm for boys and more than 88cm for females were considered high-risk [18].

Blood pressure (BP) was measured twice with a mercury sphygmomanometer, first in a sitting position and once after 15min of rest. The second BP measurement was done 5min after the first. The average value was calculated and the BP result was taken. SBP of 140 mmHg or DBP of 90 mmHg, as well as the usage of antihypertensive medicines on a regular basis, were used to identify hypertension [17].

A smoking habit was defined as smoking tobacco products on a daily basis during the data collection period. Participants who consumed alcohol and chewed khat within the previous 30 days of the data collection period were classified as current alcohol users and khat chewers, respectively.

### Data quality management

A pretest was conducted to check that the questions were appropriate for the study. An Amharic-translated version of the questionnaire was employed. Physical measurements were taken twice, and in some cases, three times, to reduce observer error in measurements and records, and data collectors were rotated to compare values. Before and after each day’s data collection, the sphygmomanometer used in the field was compared to the one used in the nearby hospital, Debre Tabor comprehensive hospital. For uniformity in reference and test readings, the glucometer gadget and strips were examined on a regular basis. Every day, the data is cleaned, coded, and recorded.

### Data analysis

The collected data was double-checked for accuracy. Completed questionnaires were entered into Epi–Info version 7.2.5.0 immediately after data collection and then exported to SPSS version 25 for analysis. Means, percentages, standard deviations, and ranges were calculated using descriptive statistics. A logistics regression was done to identify factors associated with IFG and UDM. The statistical level of significance was declared at p 0.05.

## Results

### Sociodemographic characteristics of study participants

A total of 324 participants were incorporated for the final analysis, with a response rate of 100%. The mean age of the participants was 43.76 ± 17.29 years (range 20–80 years). The bulk of the participants, 190 (58.6%) were men; 248 (76.5%) were married; 256 (79.0%) were Orthodox Christians; and 80 (24.7%) had no formal education (Table1).

**Table 1:** Sociodemographic characteristics of the study population in Woreta town, Ethiopia, 2021

Variables	Frequency	Percentage
**Sex**		
Male	190	58.6
Female	134	41.4
**Age group (years)**		
20–30	60	18.5
31–40	48	14.8
41–50	76	23.5
51–60	72	22.2
≥ 61	68	21.0
**Marital status**		
Married	248	76.5
Single	36	11.1
Divorced	12	3.7
Widowed/separated	28	8.6
**Level of education**		
Not able to read and write	80	24.7
Primary school	84	25.9
High school	36	11.1
Higher educations	124	38.3
**Religion**		
Orthodox	256	79.0
Muslim	56	17.3
Protestant	12	3.7
**Occupation**		
Farmer	60	18.5
housewife	80	24.7
Merchant	64	19.8
Employed	120	37.0
**Smoking habit**		
Yes	12	3.7
No	312	96.3
**Khat chewing**		
Yes	52	84.0
No	272	16.0
**Moderate alcohol intake**		
Yes	108	33.3
No	216	66.7
**Perform physical exercise**		
Yes	16	4.9
No	308	95.1

### Magnitude of impaired fasting glucose and undiagnosed DM

The magnitude of IFG and UDM among the study subjects were 12% (95% CI 9–16) and 2.3% (95% CI 1.1–4), respectively, according to the American Diabetes Association (ADA) fasting criteria. IFG was found to be prevalent in 19.7% of males and 23.5% of females in our study area (Table2).

**Table 2:** IFG and undiagnosed DM by socio-demographic characteristics of the study participants in Woreta town, Ethiopia, 2021

Parameter	NFG (n = 152, 46.9%)	IFG (n = 140, 43.2%)	UDM (n = 32, 10.0%)
**Sex**			
Male	96(29.6)	64(19.7)	32(10.0)
Female	56(17.3)	76(23.5)	0
**Age**			
20–30	48(14.8)	12(3.7)	0
31–40	28(8.6)	12(3.7)	8(2.5)
41–50	32(9.9)	40(12.3)	4(1.2)
51–60	24(7.4)	36(11.1)	12(3.7)
≥ 61	20(6.2)	40(12.3)	8(2.5)
**Occupation**			
Farmer	24(7.4)	36(11.1)	0
housewife	24(7.4)	56(17.3)	0
Merchant	36(11.1)	20(6.2)	8(2.5)
Employed	56(17.3)	40(12.3)	24(7.4)
**Marital status**			
Married	108(33.3)	116(35.8)	24(7.4)
Single	32(9.9)	4(1.2)	0
Divorced	4(1.2)	8(2.5)	0
Widowed/separated	8(2.5)	12(3.7)	8(2.5)
**Level of education**			
No formal education	36(11.1)	40(12.3)	4(1.2)
Primary school	36(11.1)	40(12.3)	8(2.5)
High school	24(7.4)	20(6.2)	0
Higher educations	56(17.3)	48(14.8)	20(6.2)
**Religion**			
Orthodox	124(38.3)	108(33.3)	24(7.4)
Muslim	28(8.6)	24(7.4)	4(1.2)
Protestant	0	8(2.5)	4(1.2)

IFG impaired fasting glucose, N/n number, NFG normal fasting glucose, UDM undiagnosed diabetes mellitus, % percent

### Clinical presentations of study subjects with IFG and UDM

Thirty-six (11.11%) of the subjects had an overweight BMI, whereas 88.9% of the overweight subjects had IFG. About 7.4% of the participants had examined their blood glucose level previously, and 1.2% had screened for DM within the last month. The most commonly elicited symptoms of diabetes were polyuria, polydipsia, polyphagia, and weariness, which were known by eight (2.5%) of the individuals. The prevalence of UDM was reported to be 9.9%, with a 95% confidence interval of 7.5 to 11.7. Furthermore, the magnitude of IFG was 44.14%, with a 95% confidence interval of 19.45 to 64.54 (Table3).

**Table 3:** Clinical presentation and laboratory measurements of study subjects in Woreta town, Ethiopia, 2021

Characteristics	Normal (< 100mg/dl)	IFG (100–125 mg/dl)	UDM (≥ 126 mg/dl)	Chi-Square	p-value
Ever checked your blood sugar level					
Yes	4(16.7)	4(16.7)	16(66.6)	23.48	0.000
No	148(49.3)	136(45.3)	16(5.4)		
The checked blood sugar level in the last month					
Yes	0	4(100.00)	0	1.331	0.514
No	152(47.5)	136(42.5)	32(10.0)		
Family history of DM					
Yes	8(25.0)	24(75.0)	5	0.989	0.61
No	144(55.4)	116(44.6)	27(10.9)		
Diagnosis of hypertension					
Yes	12(12.2)	62(63.3)	24(24.5)	23.158	0.000
No	140(61.9)	78(34.5)	8(3.5)		
Know symptoms of DM					
Yes	26(55.3)	13(27.7)	8(17.0)	60.256	0.000
No	126(45.5)	127(45.8)	24(8.7)		
Body mass index (BMI), kg/m^2^					
Under Weight	4(100.0)	0	0	23.387	0.001
normal	144(55.4)	96(36.9)	20(7.7)		
Over Weight	4(11.1)	32(88.9)	0		
Obese	0	12(50.0)	12(50.0)		
Perform physical exercise					
Yes	16(100.0)	0	0	4.761	0.092
No	136(44.2)	140(45.4)	32(10.4)		
Moderate alcohol intake					
Yes	44(40.7)	44(40.7)	20(18.5)	3.449	0.178
No	108(50.0)	96(44.4)	12(5.6)		

### Factors associated with IFG and UDM

Age, family history of diabetes mellitus, body mass index, waist circumference, hypertriglyceridemia, and hypertension were found to be substantially associated with the prevalence of impaired fasting glucose in the bivariate analysis (Table4).

The results of the multivariate logistic regression analysis revealed that IFG was independently associated with WC, HPN, FHDM and hypertriglyceridemia. The magnitude of IFG was 1.72 times greater in participants with high risk of WC compared to low-risk WC subjects (AOR = 1.72, 95% CI 1.23–3.14). Prediabetes was 2.35 times more common in people with hypertriglyceridemia than in people with normal TG (AOR = 2.35, 95% CI 1.41–5.43). IFG was 3.48 times more common in people with hypertension compared to people without hypertension (AOR = 3.48, 95% CI 1.35–8.89) (Table4).

**Table 4:** Bivariate and multivariate analysis of factors associated with IFG in Woreta town, Ethiopia, 2021

Variable	Category	IFG		COR(95%CI)	AOR(95%CI)
		Yes	No		
Age	20–30	12	48	1	1
	31–40	12	28	1.47(0.45–4.4.62)	0.52(0.14–2.364)
	41–50	32	40	3.98(1.36–11.02) *	2.06(0.51–7.09)
	51–60	24	36	3.32(1.09–10.27) *	1.59(0.45–5.38)
	≥ 61	20	40	2.46(0.77–7.18)	1.47(0.39–5.18)
BMI (kg/m2 )	≤ 24.9	109	135	1	1
	≥25	31	17	2.26(1.19–4.30) *	1.29(1.19–3.79) *
WC	Low risk	89	125	1	1
	High risk	51	27	2.65(1.55–4.55) **	1.72(1.23–3.14) *
TG	Normal	117	139	1	1
	High	23	13	2.10(1.02–4.33) **	2.35(1.41–5.43) **
FHDM	Yes	24	8	3.72(1.61–8.6) **	2.34(1.37–5.79)^**^
	No	116	144	1	1
HPN	Yes	62	12	9.23(4.71–18.26) **	3.48 (1.35–8.89) **
	No	78	140	1	1

AOR adjusted odds ratio, BMI body mass index, CI confidence interval, COR crude odds ratio, FHDM family history of diabetic mellitus, DM diabetes mellitus, FHDM family history of diabetes mellitus, IFG impaired fasting glucose, TG triglycerides, WC waist circumference, HPN hypertension

* P < 0.05 ** P < 0.01

## Discussion

This study revealed that the prevalence of IFG was 43%, indicating that if appropriate interventions were not made, the individuals might develop diabetes. This report was higher than a population-based study in Qatar (12.5%)[19]. In Denmark (27.1%)[20] and Bukittinggi, Indonesia (32%)[21], Uganda (3.9%)[22], and Kenya (3.1%)[1]. The probable reason could be the differences in relation to sociodemographic and living standards.

In this study, the magnitude of UDM was 9.87%. It was associated with obesity, hypertriglyceridemia, a family history of DM, and hypertension. This report was in line with reports from studies in the East Gojjam zone, northwest Ethiopia (11.5%) [23] Bahir Dar city, northwest Ethiopia (10.2%)[7], Germany (8.2%)[24] USA (11.5%)[25] Italia (10%)[26] and African urban population (8.68%)[27]. However, the magnitude of UDM in this study was higher than that of a study in Uganda (2.3%)[22] and Kenya (2.4%)[1]. The possible difference between Kenya’s study and the current study is that the current study was from a single center while Kenya’s sample was a nationally representative sample. The study in Uganda was among municipal communities with better educational status, expected to have better awareness of diabetic mellitus.

Nevertheless, this report was higher than that of a study at Koladiba (2.3%)[17], Bishoftu town (5%)[7], and Addis Ababa, Ethiopia (6.6%)[28], Teheran, Iran (5%) [29] and Qatar (5.9%)[19]. Moreover, our report was lower than those of patients with UDM and hypertension in India (15%)[30]. The probable reasons for these differences could be different study settings, lifestyles, health-seeking behavior, and practice in routine screening of diabetes and other health problems. In particular, this report was significantly higher than that of the previous study in Koladiba, a rural district in northwest Ethiopia[17]. The disparity could be due to differences in sample size.

For participants with high risk of WC, the odds of IFG was twice higher than those who have low risk of WC. This finding was in line with those of studies conducted in koladiba [17]. The probable reason could be in relation to the relation of central obesity and insulin resistance. Similarly, among those who had hypertension, the odds of getting IFG were three times higher as compared to no hypertension.

For participants with a high risk of WC, the odds of IFG were twice as high as those who had a low risk of WC. This finding was in line with those of studies conducted in Koladiba [7]. The probable reason could be in relation to the relationship between central obesity and insulin resistance. Similarly, among those who had hypertension, the odds of getting IFG were three times higher as compared to those who had normal blood pressure.

Any causal association is not estimated by the cross-sectional design of this study. Self-reported dietary information, social habits, and physical activity data may yield inaccurate estimations. The study participant’s unpredictability in obtaining a fasting state is another possible limitations in a population-based study like this study, which might easily result in an overestimation of the prevalence of impaired fasting glucose and undiagnosed diabetic mellitus.

## Conclusion

In conclusion, the magnitude of IFG and UDM was significantly high. Family history of diabetes, hypertension, WC, and TG were all associated with IFG. Regular physical activities are recommended measures for the prevention and control of diabetes mellitus among the population of Ethiopia.

## Data Availability

The datasets generated and/or analysed during the current study are not publicly available due to relevant data protection but are available from the corresponding author on reasonable request.

## Abbreviations

BMI: Body Mass Index
DM: Diabetes Mellitus
FBG: Fasting Blood Glucose
IDA: International Diabetes Association’s
IFG: Impaired Fasting Glucose
NCDs: Non-Communicable Diseases
UDM: Undiagnosed Diabetes Mellitus
WHO: World Health Organization
